# Socioeconomic inequalities in stillbirth and neonatal mortality rates: evidence on Particularly Vulnerable Tribal Groups in eastern India

**DOI:** 10.1186/s12939-022-01655-y

**Published:** 2022-05-06

**Authors:** Sophie L.P. Busch, Tanja A.J. Houweling, Hemanta Pradhan, Rajkumar Gope, Shibanand Rath, Amit Kumar, Vikash Nath, Audrey Prost, Nirmala Nair

**Affiliations:** 1 Department of Public Health, Erasmus MC, University Medical Centre Rotterdam, Rotterdam, The Netherlands; 2 Ekjut, Chakradharpur, Jharkhand India; 3 Institute for Global Health, University College London, London, UK

**Keywords:** Indigenous health, Inequalities, India, Neonatal mortality, Stillbirth, Maternity care

## Abstract

**Background:**

Tribal peoples are among the most marginalised groups worldwide. Evidence on birth outcomes in these groups is scant. We describe inequalities in Stillbirth Rate (SBR), Neonatal Mortality Rate (NMR), and uptake of maternal and newborn health services between tribal and less disadvantaged groups in eastern India, and examine the contribution of poverty and education to these inequalities.

**Methods:**

We used data from a demographic surveillance system covering a 1 million population in Jharkhand State (March 2017 – August 2019) to describe SBR, NMR, and service uptake. We used logistic regression analysis combined with Stata’s adjrr-command to estimate absolute and relative inequalities by caste/tribe (comparing Particularly Vulnerable Tribal Groups (PVTG) and other Scheduled Tribes (ST) with the less marginalised Other Backward Class (OBC)/none, using the Indian government classification), and by maternal education and household wealth.

**Results:**

PVTGs had a higher NMR (59/1000) than OBC/none (31/1000) (rate ratio (RR): 1.92, 95%CI: 1.55–2.38). This was partly explained by wealth and education, but inequalities remained large after adjustment (adjusted RR: 1.59, 95%CI: 1.28–1.98). NMR was also higher among other STs (44/1000), but disparities were smaller (RR: 1.47, 95%CI: 1.23–1.75). There was a systematic gradient in NMR by maternal education and household wealth. SBRs were only higher in poorer groups (RR_poorest vs. least poor_:1.56, 95%CI: 1.14–2.13). Uptake of facility-based services was low among PVTGs (e.g. institutional birth: 25% vs. 69% in OBC/none) and among poorer and less educated women. However, 65% of PVTG women with an institutional birth used a maternity vehicle vs. 34% among OBC/none. Visits from frontline workers (Accredited Social Health Activists [ASHAs]) were similar across groups, and ASHA accompaniment of institutional births was similar across caste/tribe groups, and higher among poorer and less educated women. Attendance in participatory women’s groups was similar across caste/tribe groups, and somewhat higher among richer and better educated women.

**Conclusions:**

PVTGs are highly disadvantaged in terms of birth outcomes. Targeted interventions that reduce geographical barriers to facility-based care and address root causes of high poverty and low education in PVTGs are a priority. For population-level impact, they are to be combined with broader policies to reduce socio-economic mortality inequalities. Community-based interventions reach disadvantaged groups and have potential to reduce the mortality gap.

**Supplementary Information:**

The online version contains supplementary material available at 10.1186/s12939-022-01655-y.

## Background

Tribal and Indigenous groups are among the most marginalised groups worldwide [1]. India has the second largest population of indigenous peoples in the world. Over 100 million people, or nearly 9% of India’s population, belong to Scheduled Tribes [2]. Scheduled Tribes have higher infant mortality rates and a lower life expectancy at birth as compared to less disadvantaged groups in India [3]. Particularly Vulnerable Tribal Groups (PVTG) consist of the most vulnerable groups among Scheduled Tribes as recognized by the Indian government. This increased vulnerability is mainly due to their loss of land and livelihood [4].

Worldwide, approximately two million infants are stillborn, and another 2.4 million die in the first month of life every year (2019 estimate) [5, 6]. India has the highest number of stillbirths and neonatal deaths worldwide [5]. Its neonatal mortality and stillbirth rates are declining, but progress is slow and uneven [7, 8]. Research on socioeconomic inequalities in neonatal mortality [9, 10] and especially stillbirth rates [11] remains scant, and evidence on birth outcomes among Indigenous and tribal populations, globally and in India, is particularly scarce [1, 12]. Measuring neonatal and stillbirth rates without a reliable vital registration system is difficult, and is ideally done using prospective surveillance.

Facility-based care, such as antenatal care (ANC) and giving birth at a health facility is needed to reduce neonatal mortality. However, socioeconomic inequalities in facility-based care remain large [13]. Community-based services and interventions may link marginalised groups, such as PVTGs, with facility-based care. Although community-based interventions, such as community health worker visits and participatory women’s groups, tend to reach all socioeconomic groups [14], there is little evidence on whether these interventions reach tribal groups, including PVTG [15].

Our study aims to describe socioeconomic inequalities in stillbirth and neonatal mortality rates, and the uptake of facility and community-based maternal and newborn care services, comparing PVTGs and other Scheduled Tribes with less disadvantaged groups in India. We examine these inequalities in the context of population-wide disparities in these outcomes by wealth and maternal education, and examine to what extent inequalities between PVTG/other Scheduled Tribes and less disadvantaged groups are explained by inequalities in maternal education and wealth.

## Methods

### Study population

Our study was situated in six districts (Bokaro, Dumka, Palamu, Khunti, Ranchi and West Singhbhum) of Jharkhand State, Eastern India. Jharkhand is a primarily rural state, and 26% of its population belongs to a Scheduled Tribe [2]. Availability of health care facilities in the State remains limited compared with many other Indian States [16]. A small proportion of Jharkhand’s Scheduled Tribes population consists of PVTGs (5.7% of the Scheduled Tribes population in Jharkhand, 1.5% of the total population [17]). In order to be classified as a PVTG by the Indian government, a tribe must have the following five characteristics: “a forest-dependent livelihood; a pre-agricultural level of existence; a stagnant or declining population; low literacy rates; subsistence-based economy” [4].

In the six study districts, data were collected in a total of 20 blocks (administrative units of approximately 100,000 people), and within each block, in five clusters of approximately 10,000 people each. In total, data were collected in 100 clusters, comprising an estimated total study population of around 1,039,000 people. The six study districts were part of a non-randomised, controlled evaluation of the scale-up of a participatory women’s group intervention [18].

For our study, we used the data from the evaluation’s three control areas: 50 purposively selected geographical clusters in 10 blocks of Bokaro, Dumka and Palamu districts. We included all women who gave birth in these control clusters during the baseline (1 March 2017–31 August 2017) and evaluation period (1 September 2017–31 August 2019) of the intervention. For one outcome (attendance of women’s group meetings), we used data from the three intervention districts (Khunti, Ranchi and West Singhbhum) during the intervention period, because this is where and when the women’s group intervention was implemented.

### Data collection

In the study area, a prospective surveillance system was established for the evaluation of the scale-up of a participatory women’s group intervention [18, 19]. Local key informants (lay female community members) were incentivized to record all births and maternal and newborn infant deaths in the study clusters. Full-time paid interviewers verified the information provided by key informants. Women with a verified delivery were approached for an interview approximately six weeks after giving birth. During these interviews (conducted at the homes/compounds of the women), information was collected on vital events (births, stillbirths, neonatal and maternal deaths), use of facility- and community-based care, practices during pregnancy, delivery, and postnatal period, as well as information on sociodemographic characteristics.

### Outcomes

The main outcome of our study was neonatal mortality (deaths in the first 28 days of life among live births) (overview of indicators in Table 1). We also performed analyses for stillbirths, deaths on the first day of life, early neonatal mortality (deaths on day 1–7 of life) and late neonatal mortality (deaths on day 8–28). Our mortality analyses excluded six undetermined cases, i.e. when it was impossible to determine whether a death was a stillbirth or a neonatal death. We conducted a sensitivity analysis by including these six cases (other Scheduled Tribes (ST) one death, Scheduled Castes (SC) one death, and Other Backward Class (OBC)/none four deaths) as stillbirth, and, subsequently, as neonatal death on day 1. To explore potential explanations for our mortality findings and entry points for intervention, we also performed analyses regarding the uptake of community and facility-based maternal and newborn care services.

**Table 1:** Definition of outcomes

**MORTALITY OUTCOMES**	
Neonatal mortality rate	Deaths during the first 28 days of life/1000 livebirths
Mortality rate on day 1	Deaths on day 1 of life/1000 livebirths
Early neonatal mortality rate	Deaths on day 1-7 of life/1000 livebirths
Late neonatal mortality rate	Deaths on day 8-28 of life/1000 livebirths
Stillbirth rate	Stillbirths/1000 births
**FACILITY-BASED CARE & SERVICES**	
≥3 ANC	Mothers who had at least three antenatal care check-ups (all women who gave birth)
Birth plan	Mothers who made a plan for all of the following: place of delivery, delivery attendant, money, transport, safe delivery kit (all women who gave birth)
Care for problem in pregnancy	Mothers who sought care from a skilled attendant (Auxiliary Nurse Midwife (ANM) or doctor) for a problem during their pregnancy (all women who had a health problem during pregnancy)
Maternity vehicle (*mamta vahan*)^a^	Mothers who used a maternity vehicle for transport during delivery (all women who had an institutional birth)
Institutional birth	Mothers who gave birth at a health care facility (private/public) (all women who gave birth)
JSY cash incentive	Mothers who received money from the Janani Suraksha Yojani (JSY)-scheme (all women who had an institutional birth). The JSY cash transfer scheme is an intervention set up by the National Health Mission of the Indian Government. The scheme aims to reduce neonatal and maternal mortality by providing cash incentives for marginalised mothers who have an institutional birth [20].
Postnatal care for the mother	Mothers who received postnatal care for themselves by a skilled attendant (ANM, doctor, nurse) within six weeks after delivery (all women who gave birth)
Care for neonatal problems	Mothers who sought care from an ANM, Anganwadi Worker (AWW), Accredited Social Health Activist (ASHA) (community health worker) or a doctor for a health problem of their baby (all infants who had a health problem in the neonatal period)
**COMMUNITY-BASED INTERVENTIONS**	
Women’s group meeting attendance	Mothers who attended at least one participatory women’s group meeting (all women who gave birth). The women’s group meetings were organized as a participatory learning and action intervention, facilitated by ASHAs [18]. The women’s groups met every month under the guidance of the facilitator. In a cycle of meetings, they identified and prioritized maternal and newborn health problems, identified and planned strategies to address these problems, then implemented these strategies with the support of the community, and then evaluated their strategies.
Accompanied by an ASHA	Mothers who were accompanied to a health care facility for their delivery by an ASHA (all women who gave birth)
Any ASHA visit	Mothers who received at least one postnatal ASHA-visit within the first week after delivery (all women who gave birth)
≥3 ASHA visits	Mothers who received at least three ASHA visits within the first week after delivery as recommended by the Home-Based Newborn Care of the Rural Health Mission [21] (all women who gave birth)
Support from an ASHA	Mothers who received support during their pregnancy, delivery and/or postnatal period from an ASHA (all women who gave birth)

^a^ Maternity vehicles are provided by the health centre, but called by the community, so could be considered a combined facility and community-based service

### Indicators of socioeconomic position

We used indicators of socioeconomic position that are relevant to the rural Indian context: caste/tribal affiliation, maternal education, and household wealth. For caste/tribal affiliation we distinguished ST, SC, and OBC/none, using the classification of the Indian Government. ST, SC, and OBC are all historically disadvantaged groups as recognised by the Indian Government [22]. Within these, STs, and especially PVTGs, are most marginalised, and OBC comparatively less marginalised. The following tribes in our study population are recognized as PVTG: Asurs, Savar, Mal Paharia, Sauria Paharia, Paharia, Hill Kharia, Kumar Bhag Paharia [23]. The population belonging to neither ST, SC or OBC was too small to study separately (around 1%), and was therefore combined with the OBC. We compared outcomes of PVTGs and other STs with OBC/none.

We measured maternal education using the number of years of school attendance. Based on this, women were categorized as follows: had not attended school, primary education (< 7 years of education, secondary education (8–10 years) and higher secondary and above (11 + years). We measured wealth using asset ownership (bed/cot, chair, table, pressure-cooker, electricity, fan, radio/tap recorder, TV, watch/clock, telephone/mobile, bicycle, motorcycle, fridge, computer/tablet, tractor, lift pump). We constructed a wealth index for the full study population in the six districts using a principal component analysis (PCA), and subsequently divided the respondents into wealth quintiles [24]. We did so separately for the baseline and evaluation period, given potential population level changes in asset ownership over time.

### Statistical analysis

We calculated stillbirth (SBR) and neonatal mortality rates (NMR) and uptake of services for each dimension of socioeconomic position. We then conducted univariable logistic regression analyses to estimate the magnitude of socioeconomic inequalities in these outcomes. Then, using multivariable logistic regression analyses, we examined if socioeconomic inequalities in mortality and service uptake were explained by maternal age at childbirth and parity. This is important, because maternal age at childbirth and parity are strongly associated with socioeconomic position (SEP) in our study population, and they are important determinants of the outcomes under study. Finally, we examined whether inequalities by caste/tribal affiliation were explained by maternal education and wealth, by adding these to the regression models. To fully adjust for maternal education and wealth, we included these as continuous variables (education in years, wealth as PCA scores). Relative inequalities are presented as rate ratios (RR) and absolute inequalities as rate differences (RD). Both were calculated using Stata’s adjrr-command [25], which can be used to calculate RRs and RDs from logistic regression models.

We used Stata’s svy-command to adjust for the two-stage sampling design with stratification: the first stage of sampling was done at the block level – in which blocks were sampled in a stratified manner by district (i.e. districts were defined as strata) - and clusters were sampled within blocks [26, 27]. Since over 5% of the total population was sampled, we applied a finite population correction. Stata (version 15.1) was used for all analyses [28].

## Results

A total of 24,984 women gave birth in the three control districts (Table 2). Almost half of this population (48%) belonged to a ST. Of these, a minority belonged to one of the PVTGs (4% of the total population). 40% of the population belonged to the OBC/none category. PVTGs were highly socioeconomically deprived, much more so than STs. Over half (58%) of women in the PVTG group had never attended school and 68% belonged to the poorest quintile, compared with 33% in the ST group and with 20% and 9% respectively in the OBC/none category.

**Table 2:** Socioeconomic and demographic characteristics of the study population^a^

	In category		Age distribution within categories (%)					Parity distribution within categories (%)		Education distribution within categories (%)				Wealth distribution within categories (%)				
	*n*^b^	%	<20 years (*n* = 2985)	20-24 years (*n* = 11655)	25-29 years (*n* = 7240)	30-34 years (*n* = 2150)	35+ years (*n* = 827)	Primi-parity (*n* = 7638)	Multi-parity (*n* = 17278)	Has not attended school (*n* = 7149)	Primary school (*n* = 7247)	Secon-dary school (*n* = 7368)	Higher secon-dary + (*n* = 3152)	Poorest (*n* = 5696)	Next poor (*n* = 5441)	Middle poor (*n* = 5487)	Less poor (*n* = 4954)	Least poor (*n* = 3406)
**Total population**	24984	100	12	47	29	9	3	31	69	29	29	30	13	23	22	22	20	14
**Tribe/caste of the mother**^**c**^																		
ST	12104	48	12	43	30	11	4	31	69	33	34	26	8	33	26	21	13	6
PVTG	1026	4	11	37	34	12	6	25	75	58	33	8	1	68	20	7	3	1
Other ST	11078	44	12	44	30	11	4	32	68	31	34	27	8	30	26	22	14	7
SC	2996	12	12	50	29	7	3	29	71	40	23	26	11	25	25	22	16	12
OBC/none	9884	40	13	51	28	7	2	31	69	20	25	35	19	9	16	23	29	23
**Maternal education**																		
Has not attended school	7149	29	5	32	38	17	7	16	84					40	26	20	11	3
Primary school	7247	29	12	42	34	9	3	26	74					27	27	24	17	6
Secondary school	7368	30	20	58	19	3	1	42	58					11	19	25	27	18
Higher secondary & above	3152	13	11	66	19	2	0	49	51					3	8	16	29	45
**Household wealth**																		
Poorest	5696	23	10	36	34	14	6	22	78	50	34	14	1					
Next-poor	5441	22	12	43	32	9	4	29	71	34	36	25	4					
Middle poor	5487	22	12	49	28	8	3	32	68	26	31	34	9					
Less poor	4954	20	14	53	25	7	2	35	65	16	24	41	19					
Least poor	3406	14	13	58	24	4	1	39	61	6	13	40	41					

^a^ This table gives the distribution of women who have given birth in the three control districts during the study period

^b^ n varies due to missing values

^**c**^ *ST* Scheduled Tribes, *PVTG* Particularly Vulnerable Tribal Groups, *Other ST* Other Scheduled Tribes, *SC* Scheduled Caste, *OBC/none* Other Backwards Class or none of the above

### Stillbirth and neonatal mortality rates

The SBR in our total study population was 22 per 1000 births (567 stillbirths in 25,227 births) (Table 3 and Supplementary Table 1). Unadjusted SBRs were similar between PVTG and OBC/none (RR: 0.75, 95%CI: 0.47–1.2) and between ST and OBC/none (RR: 0.88, 95%CI 0.74; 1.06). After adjustment for age, parity, maternal education, and wealth, SBRs were lower among PVTG and ST compared with OBC/none (PVTG vs. OBC: RR: 0.54, 95% CI: 0.33–088; ST vs. OBC: RR: 0.74, 95% CI: 0.62–0.89). Conversely, poverty was systematically associated with higher SBRs (poorest vs. richest: RR: 1.56, 95%CI 1.14–2.13), which was for a minor part explained by age and parity (RR: 1.50, 95% 1.08–2.09). There were no statistically significant differences in SBR between educational groups.

**Table 3:** Socioeconomic inequalities in stillbirth rate and neonatal mortality rate

	Stillbirths rate per 1000 births and inequalities		Neonatal mortality rate per 1000 live births and inequalities		Mortality on day 1 rate per 1000 live births and inequalities		Early neonatal mortality (day 1-7) rate per 1000 live births and inequalities		Late neonatal mortality (day 8-28) rate per 1000 live births and inequalities	
**Total Population**	22		39		17		31		8	
**Tribe/caste of the mother**^**a**^										
ST	21		45		18		35		10	
PVTG	17		59		24		43		16	
Other ST	21		44		17		35		9	
SC	28		42		22		35		7	
OBC/none	23		31		14		25		5	
*PVTG vs. OBC/none*										
RD^b^ (95% CI)	-6	(-14; 3)	28	(19; 38)	10	(1; 18)	18	(8; 28)	10	(5; 16)
RD adjusted for age & parity (95% CI)	-7	(-15; 0)	29	(20; 38)	10	(2; 18)	19	(9; 29)	10	(4; 16)
RD adjusted for wealth, education, age & parity (95% CI)	-12	(-19; -4)	19	(10; 29)	7	(-1; 14)	12	(2; 22)	7	(2; 12)
RR^c^ (95% CI)	0.75	(0.47; 1.2)	1.92	(1.55; 2.38)	1.67	(1.08; 2.57)	1.70	(1.3; 2.23)	2.95	(1.87; 4.65)
RR adjusted for age & parity (95% CI)	0.69	(0.43; 1.09)	1.94	(1.58; 2.37)	1.71	(1.13; 2.58)	1.73	(1.34; 2.24)	2.87	(1.77; 4.67)
RR adjusted for wealth, education, age & parity (95% CI)	0.54	(0.33; 0.88)	1.59	(1.28; 1.98)	1.45	(0.96; 2.18)	1.43	(1.07; 1.92)	2.26	(1.42; 3.58)
*ST vs. OBC/none*										
RD (95% CI)	-3	(-7; 1)	14	(9; 20)	4	(-1; 8)	10	(5; 15)	5	(2; 7)
RD adjusted for age & parity (95% CI)	-4	(-7; 0)	14	(8; 20)	4	(-1; 8)	10	(4; 15)	4	(2; 7)
RD adjusted for wealth, education, age & parity (95% CI)	-7	(-11; -3)	10	(4; 16)	2	(-2; 7)	7	(2; 12)	3	(1; 6)
RR (95% CI)	0.88	(0.74; 1.06)	1.47	(1.23; 1.75)	1.25	(0.91; 1.72)	1.39	(1.15; 1.67)	1.84	(1.31; 2.59)
RR adjusted for age & parity (95% CI)	0.85	(0.72; 1)	1.45	(1.22; 1.72)	1.25	(0.92; 1.7)	1.37	(1.14; 1.65)	1.79	(1.26; 2.54)
RR adjusted for wealth, education, age & parity (95% CI)	0.74	(0.62; 0.89)	1.31	(1.1; 1.55)	1.16	(0.85; 1.58)	1.25	(1.05; 1.5)	1.57	(1.12; 2.2)
**Maternal education**										
Has not attended school	25		45		20		35		10	
Primary education	23		40		16		32		8	
Secondary education	19		34		15		29		5	
Higher secondary & above	20		33		15		26		7	
*Lowest vs. highest education level*										
RD (95% CI)	5	(-1; 11)	13	(3; 22)	5	(-1; 11)	9	(1; 17)	3	(-1; 8)
RD adjusted for age & parity (95% CI)	5	(-1; 11)	19	(9; 29)	8	(1; 15)	15	(7; 23)	4	(-1; 8)
RR (95% CI)	1.26	(0.94; 1.69)	1.38	(1.04; 1.84)	1.33	(0.9; 1.98)	1.36	(1.01; 1.83)	1.49	(0.88; 2.52)
RR adjusted for age & parity (95% CI)	1.25	(0.94; 1.64)	1.61	(1.21; 2.14)	1.55	(1.02; 2.36)	1.61	(1.18; 2.2)	1.60	(0.96; 2.65)
**Household wealth**										
Poorest	26		47		18		36		11	
Next poor	27		47		22		38		10	
Middle poor	19		33		15		27		6	
Less poor	22		33		15		28		5	
Least poor	17		32		14		25		6	
*Poorest vs. least poor*										
RD (95% CI)	9	(3; 16)	15	(7; 23)	4	(-3; 10)	10	(4; 17)	5	(1; 8)
RD adjusted for age & parity (95% CI)	8	(2; 15)	17	(8; 25)	4	(-2; 10)	12	(6; 19)	5	(1; 9)
RR (95% CI)	1.56	(1.14; 2.13)	1.47	(1.13; 1.9)	1.25	(0.8; 1.94)	1.41	(1.11; 1.79)	1.70	(1.09; 2.67)
RR adjusted for age & parity (95% CI)	1.50	(1.08; 2.09)	1.54	(1.2; 1.97)	1.30	(0.85; 2.01)	1.49	(1.18; 1.88)	1.71	(1.08; 2.71)

^a^ *ST* Scheduled Tribes, *PVTG* Particularly Vulnerable Tribal Groups, *Other ST* Other Scheduled Tribes, *SC* Scheduled Caste, *OBC/none* Other Backwards Class or none of the above

^b^ *RD* Rate Difference

^c^ *RR* Rate Ratio

**Fig. 1:**
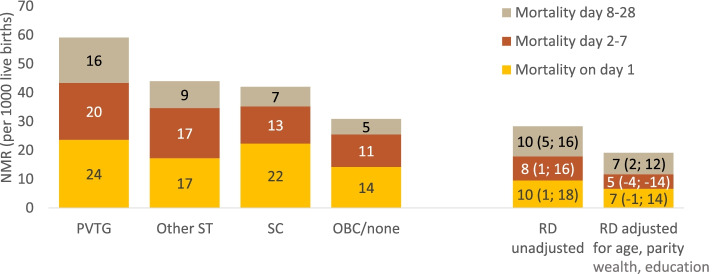
Neonatal mortality by tribal/caste affiliation of the mother and day of death. PVTG = Particularly Vulnerable Tribal Groups, Other ST = Other Scheduled Tribes, SC = Scheduled Caste, OBC/none = Other Backwards Class or none of the above, RD = Rate Difference

The NMR in our total study population was 39/1000 livebirths (966/24,660). Nearly 80% of neonatal deaths occurred in the first week of life (31/1000), of which over half occurred on day one (17/1000). PVTG had a very high NMR (59/1000), whereas OBC/none had the lowest NMR (31/1000) (RD: 28, 95% CI: 19–38, RR: 1.92, 95% CI: 1.55–2.38). Other STs had an NMR that was comparable to SC (44 and 42/1000 respectively), but higher than OBC/none (RR: 1.47, 95%CI: 1.23–1.75). Roughly two thirds of the absolute inequalities in NMR between PVTG and OBC/none consisted of early neonatal deaths (one third on day 1 and one third on day 2–7) (Fig. 1). These inequalities remained largely similar after adjustment for age and parity. The higher NMR among PVTG was only partly explained by their higher levels of poverty and lower levels of maternal education, with large inequalities remaining after adjustment (adjusted RR: 1.59, 95% CI: 1.28–1.98).

Less educated and poorer groups also had systematically higher NMRs compared with better-off peers (no schooling vs. higher secondary: RR: 1.38, 95% CI: 1.04–1.84; poorest vs. least poor: RR: 1.47, 95% CI: 1.13–1.90). These mortality inequalities would have been somewhat larger if it had not been for the more favourable demographic characteristics (especially multiparity) of the lower SEP groups (adjusted RR, no schooling vs. higher secondary: 1.61, 95% CI: 1.21–2.14; poorest vs. least poor: 1.54, 95% CI: 1.20–1.97) (Tables 2 and 3, Supplementary Table 2).

The sensitivity analyses that included the six undetermined deaths showed virtually identical results as the analyses that excluded these cases (results not shown).

### Facility-based services

Uptake of facility-based services was generally low (Table 4). Around one quarter of mothers (27%) had received three or more antenatal visits and 55% had an institutional birth. Only 41% of women sought care for problems during pregnancy, whereas care for neonatal problems was higher (63%). Over 40% of women who had an institutional birth were transported by a maternity vehicle (*mamta vahan*), and only a minority (6%) had received the Janani Suraksha Yojani (JSY) cash incentive for institutional birth at the time of the interview.

**Table 4:** Socioeconomic inequalities in the uptake of facility-based care

	≥3 ANC % and inequalities	Birth plan % and inequalities	Care for problem in pregnancy^**a**^ % and inequalities	Maternity vehicle﻿^**b**^ % and inequalities	Institutional birth % and inequalities	JSY cash incentive^**b**^ % and inequalities	Postnatal care for the mother % and inequalities	Care for neonatal problems^**c**^ % and inequalities
**Total population**	27	32	41	41	55	6	22	63
**Tribe/caste**^**d**^								
ST	21	29	35	51	44	5	18	58
PVTG	10	13	19	65	25	4	8	36
Other ST	22	30	36	50	46	5	19	59
SC	28	29	40	34	52	8	22	61
OBC & none	35	37	49	34	69	7	28	70
*PVTG vs. OBC/none*								
RD^e^ (95% CI)	-25 (-33; -17)	-24 (-37; -10)	-30 (-40; -20)	30 (19; 41)	-43 (-53; -33)	-3 (-6; 0)	-19 (-30; -9)	-34 (-48; -20)
RD adjusted for age & parity (95% CI)	-25 (-33; -16)	-23 (-36; -10)	-30 (-40; -20)	29 (18; 41)	-42 (-52; -32)	-3 (-6; 1)	-19 (-30; -8)	-33 (-47; -20)
RD adjusted for wealth, education, age & parity (95% CI)	-14 (-22; -6)	-11 (-26; 3)	-26 (-36; -16)	22 (9; 36)	-24 (-35; -14)	-2 (-5; 2)	-17 (-27; -6)	-21 (-36; -6)
RR^f^ (95% CI)	0.28 (0.16; 0.48)	0.35 (0.14; 0.89)	0.38 (0.27; 0.55)	1.88 (1.45; 2.45)	0.37 (0.25; 0.53)	0.58 (0.29; 1.19)	0.3 (0.16; 0.56)	0.52 (0.42; 0.63)
RR adjusted for age & parity (95% CI)	0.29 (0.17; 0.51)	0.37 (0.15; 0.92)	0.38 (0.27; 0.55)	1.86 (1.43; 2.42)	0.39 (0.27; 0.56)	0.6 (0.29; 1.24)	0.31 (0.16; 0.58)	0.53 (0.43; 0.64)
RR adjusted for wealth, education, age & parity (95% CI)	0.54 (0.35; 0.84)	0.65 (0.32; 1.32)	0.45 (0.31; 0.65)	1.62 (1.2; 2.19)	0.61 (0.47; 0.8)	0.71 (0.34; 1.47)	0.36 (0.2; 0.67)	0.69 (0.55; 0.86)
*ST vs. OBC/none*								
RD (95% CI)	-14 (-20; -7)	-8 (-17; 2)	-14 (-23; -5)	16 (9; 24)	-24 (-32; -16)	-2 (-4; 0)	-10 (-18; -2)	-13 (-26; 0)
RD adjusted for age & parity (95% CI)	-13 (-20; -7)	-7 (-16; 2)	-14 (-22; -6)	16 (9; 24)	-24(-31; -16)	-2 (-4; 0)	-10 (-18; -2)	-13 (-25; 0)
RD adjusted for wealth, education, age & parity (95% CI)	-6 (-11; -1)	1 (-6; 8)	-11 (-19; -4)	12 (3; 21)	-14 (-20; -8)	-1 (-3; 1)	-8 (-15; -1)	-7 (-20; 7)
RR (95% CI)	0.6 (0.48; 0.75)	0.79 (0.58; 1.08)	0.71 (0.58; 0.89)	1.47 (1.23; 1.76)	0.65 (0.55; 0.76)	0.75 (0.56; 1.01)	0.65 (0.47; 0.89)	0.82 (0.68; 0.99)
RR adjusted for age & parity (95% CI)	0.61 (0.49; 0.76)	0.81 (0.6; 1.09)	0.71 (0.58; 0.88)	1.47 (1.23; 1.76)	0.66 (0.56; 0.76)	0.76 (0.57; 1.03)	0.65 (0.47; 0.89)	0.82 (0.68; 0.98)
RR adjusted for wealth, education, age & parity (95% CI)	0.8 (0.67; 0.96)	1.03 (0.83; 1.28)	0.76 (0.63; 0.92)	1.34 (1.09; 1.65)	0.77 (0.69; 0.87)	0.82 (0.61; 1.11)	0.71 (0.53; 0.95)	0.9 (0.74; 1.1)
**Maternal education**								
Has not attended school	17	22	33	39	38	6	19	56
Primary school	22	28	41	48	49	5	19	57
Secondary school	33	38	45	42	66	7	25	69
Higher secondary & above	48	49	52	30	83	7	29	80
*Lowest vs. highest education level*								
RD unadjusted (95% CI)	-31 (-39; -23)	-27 (-36; -19)	-18 (-26; -10)	9 (6; 12)	-45 (-52; -39)	-1 (-3; 0)	-10 (-15; -6)	-23 (-32; -15)
RD adjusted for age & parity (95% CI)	-30 (-39; -22)	-27 (-36; -17)	-18 (-26; -11)	19 (14; 24)	-42 (-49; -35)	-3 (-5; 0)	-9 (-14; -5)	-22 (-31; -13)
RR unadjusted (95% CI)	0.36 (0.27; 0.48)	0.44 (0.35; 0.56)	0.64 (0.54; 0.77)	1.32 (1.18; 1.47)	0.45 (0.38; 0.54)	0.8 (0.65; 1)	0.64 (0.5; 0.83)	0.71 (0.59; 0.85)
RR adjusted for age & parity (95% CI)	0.36 (0.26; 0.5)	0.46 (0.36; 0.58)	0.65 (0.54; 0.77)	1.64 (1.42; 1.9)	0.48 (0.41; 0.57)	0.65 (0.48; 0.88)	0.67 (0.52; 0.88)	0.72 (0.59; 0.87)
**Household wealth**								
Poorest	15	19	30	50	36	5	15	48
Less poor	22	26	40	48	48	6	21	57
Middle poor	27	32	45	42	56	6	24	68
Less poor	34	42	47	36	67	7	26	71
Least poor	48	50	47	30	80	7	26	78
*Poorest vs. least poor*								
RD unadjusted (95% CI)	-34 (-42; -26)	-31 (-44; -19)	-18 (-30; -5)	20 (14; 27)	-44 (-51; -37)	-3 (-5; -1)	-11 (-19; -3)	-30 (-38; -21)
RD adjusted for age & parity (95% CI)	-33 (-41; -25)	-30 (-43; -17)	-17 (-30; -4)	20 (14; 27)	-41 (-48; -34)	-3 (-5; -1)	-10 (-19; -2)	-29 (-38; -20)
RR unadjusted (95% CI)	0.3 (0.23; 0.39)	0.37 (0.24; 0.59)	0.63 (0.45; 0.89)	1.66 (1.34; 2.06)	0.45 (0.37; 0.54)	0.64 (0.48; 0.85)	0.57 (0.4; 0.82)	0.62 (0.51; 0.75)
RR adjusted for age & parity (95% CI)	0.31 (0.24; 0.42)	0.39 (0.25; 0.61)	0.64 (0.45; 0.91)	1.66 (1.36; 2.04)	0.48 (0.4; 0.57)	0.63 (0.48; 0.83)	0.59 (0.4; 0.88)	0.63 (0.52; 0.76)

^a^ Denominator: all women who had a health problem during pregnancy

^b^ Denominator: all women who had an institutional birth

^c^ Denominator: all infants who had a health problem in the neonatal period

^d^ *ST* Scheduled Tribes, *PVTG* Particularly Vulnerable Tribal Groups, *Other ST* Other Scheduled Tribes, *SC* Scheduled Caste, *OBC/none* Other Backwards Class or other than the above

^e^ *RD* Rate Difference

^f^ *RR* Rate ratio

PVTGs had much lower uptake of facility-based services than the other groups. The contrast with the OBC/none group was particularly large, with rate differences ranging between twenty to over 40% points for most services. For example, only 10% of PVTG women had received at least 3 ANC visits and 25% had an institutional birth, compared with 35% and 69% respectively in the OBC/none category (RD for ≥ 3 ANC: -25, 95%CI: -33 - -17; RD for institutional birth: -43, 95%CI: -53 – -33). Use of a maternity vehicle was an exception: 65% of PVTG women who had an institutional birth used this service, compared with 34% in the OBC/none group. Age and parity did not explain these inequalities in a multivariable analysis. Further adjustment by wealth and maternal education attenuated these inequalities, but uptake of facility-based services remained substantially lower in the PVTG group than in the OBC/none group (e.g. adjusted RD institutional birth: -24, 95% CI: -35 - -14). Inequalities in facility-based services between other ST and OBC/none also remained substantial, but were much smaller than those observed for PVTG (e.g. adjusted RD institutional birth: -14, 95% CI: -20 - -8).

Inequalities by maternal education and wealth followed a similar pattern: women with lower educational attainment and living in poorer households showed much lower use of facility-based services. Use of a maternity vehicle, again, was the exception, with higher use among more disadvantaged groups. Socioeconomic differences in age and parity did not explain these inequalities in service use. Use of a maternity vehicle became more pro-equitable, when using maternal education as SEP indicator, after taking differences in age and parity into account.

### Community-based services and interventions

The uptake of community-based services and interventions seemed generally higher than for facility-based services (Table 5). Nearly half (46%) of pregnant women had attended participatory women’s group meetings. Two-thirds of women with an institutional birth were accompanied by an Accredited Social Health Activist (ASHA, community health worker), and 58% of women had a postnatal ASHA visit within the first seven days after birth. Nevertheless, only 11% had the recommended three or more ASHA visits within the first week. ASHAs were mentioned as a source of support during the maternity and newborn period by 42% of women.

**Table 5:** Socioeconomic inequalities in the uptake of community-based interventions

	WGM attendance^**a**^ % and inequalities	Accompanied by ASHA^**b**^ % and inequalities	Any ASHA visit % and inequalities	≥3ASHA visits % and inequalities	ASHA Support % and inequalities
**Total population**	46	64	58	11	42
**Tribe/caste**^**c**^					
ST	43	70	61	13	41
PVTG﻿	--^d^	67	49	9	26
Other ST	43	70	62	13	43
SC	47	65	52	9	40
OBC & none	52	60	55	11	44
*PVTG vs. OBC/none*					
RD^e^ (95% CI)	--	7 (-4; 17)	-6 (-14; 2)	-2 (-6; 2)	-18 (-37; 0)
RD adjusted for age & parity (95% CI)	--	7 (-3; 18)	-6 (-14; 2)	-2 (-6; 2)	-17 (-36; 1)
RD adjusted for wealth, education, age & parity (95% CI)	--	3 (-8; 13)	-3 (-10; 5)	-1 (-5; 3)	-7 (-24; 10)
RR^f^ (95% CI)	--	1.11 (0.95; 1.31)	0.9 (0.77; 1.05)	0.82 (0.53; 1.27)	0.59 (0.29; 1.18)
RR adjusted for age & parity (95% CI)	--	1.12 (0.96; 1.31)	0.9 (0.77; 1.05)	0.81 (0.53; 1.26)	0.61 (0.3; 1.21)
RR adjusted for wealth, education, age & parity (95% CI)	--	1.04 (0.88; 1.23)	0.95 (0.83; 1.09)	0.9 (0.59; 1.36)	0.83 (0.5; 1.39)
*ST vs. OBC/none*					
RD (95% CI)	-8 (-14; -3)	10 (5; 16)	6 (0; 12)	2 (-2; 5)	-3 (-14; 8)
RD adjusted for age & parity (95% CI)	-8 (-13; -3)	10 (5; 16)	6 (0; 12)	2 (-2; 5)	-2 (-14; 9)
RD adjusted for wealth, education, age & parity (95% CI)	-5 (-10; 0)	8 (1; 15)	8 (2; 14)	2 (-1; 6)	4 (-5; 13)
RR (95% CI)	0.84 (0.74; 0.95)	1.17 (1.07; 1.28)	1.12 (1; 1.25)	1.16 (0.87; 1.54)	0.93 (0.71; 1.22)
RR adjusted for age & parity (95% CI)	0.85 (0.75; 0.95)	1.17 (1.07; 1.28)	1.12 (1; 1.25)	1.16 (0.87; 1.54)	0.94 (0.73; 1.23)
RR adjusted for wealth, education, age & parity (95% CI)	0.89 (0.8; 1)	1.13 (1.02; 1.26)	1.15 (1.03; 1.28)	1.22 (0.91; 1.63)	1.1 (0.88; 1.37)
**Maternal education**					
Has not attended school	40	68	54	11	34
Primary school	46	67	59	11	41
Secondary school	51	65	60	11	48
Higher secondary & above	47	56	58	14	51
*Lowest vs. highest education level*					
RD unadjusted (95% CI)	-7 (-13; -1)	12 (6; 19)	-4 (-10; 2)	-3 (-6; 1)	-18 (-26; -9)
RD adjusted for age & parity (95% CI)	-9 (-16; -3)	12 (5; 19)	-4 (-10; 2)	-3 (-7; 1)	-16 (-25; -8)
RR unadjusted (95% CI)	0.85 (0.74; 0.99)	1.22 (1.09; 1.37)	0.93 (0.84; 1.03)	0.8 (0.61; 1.05)	0.66 (0.54; 0.81)
RR adjusted for age & parity (95% CI)	0.81 (0.69; 0.94)	1.21 (1.07; 1.38)	0.93 (0.83; 1.03)	0.77 (0.57; 1.04)	0.68 (0.55; 0.84)
**Household wealth**					
Poorest	38	69	55	11	30
Less poor	45	68	58	11	39
Middle poor	46	66	59	11	45
Less poor	51	64	58	11	50
Least poor	50	57	59	13	54
*Poorest vs. least poor*					
RD unadjusted (95% CI)	-12 (-17; -7)	13 (4; 22)	-4 (-11; 3)	-1 (-5; 3)	-24 (-38; -10)
RD adjusted for age & parity (95% CI)	-13 (-18; -8)	13 (4; 22)	-4 (-11; 3)	-1 (-6; 3)	-23 (-37; -9)
RR unadjusted (95% CI)	0.75 (0.67; 0.85)	1.22 (1.05; 1.43)	0.93 (0.83; 1.05)	0.89 (0.63; 1.26)	0.55 (0.38; 0.8)
RR adjusted for age & parity (95% CI)	0.74 (0.66; 0.84)	1.23 (1.05; 1.43)	0.94 (0.83; 1.05)	0.88 (0.63; 1.24)	0.56 (0.39; 0.82)

^a^ Analyses for women’s group meeting attendance were performed for all mothers who gave birth in the intervention districts during the intervention period (*n* = 18726)

^b^ Denominator: women who had an institutional birth

^c^ *ST* Scheduled Tribes, *PVTG* Particularly Vulnerable Tribal Groups, *Other ST* Other Scheduled Tribes, *SC* Scheduled Caste, *OBC/none* Other Backwards Class or other than the above

^d^ Since only five PVTG women gave birth in the intervention period in the intervention areas, we were not able to reliably estimate women’s group attendance for PVTGs. Four out of five PVTG-women who gave birth in the intervention areas during the intervention period attended women’s group meetings

^e^ *RD* Rate Difference

^f^ *RR* Rate Ratio

Socioeconomic inequalities in community-based interventions were smaller than those for facility-based services. Women’s group attendance was between 40 and 50% across all socioeconomic categories, with small differences between ST and OBC/none, which disappeared after adjustment for age, parity, education, and wealth. There was a weak gradient in women’s group attendance by maternal education and wealth, with somewhat lower attendance among poorer and less educated groups.

Uptake of services provided by ASHAs was generally equitable. ASHAs were as likely to accompany PVTG as OBC/none women for institutional birth (RR: 1.11, 95% CI: 0.95–1.31), and more likely to accompany ST, poorer and less educated women compared with better-off groups. There were no detectable differences in any postnatal ASHA visit between PVTG and OBC/none (RR: 0.90, 95% CI: 0.77–1.05), nor across education and wealth groups, whereas such visits were slightly more common in STs. Receipt of ≥ 3 ASHA visits was comparably low across all groups. ASHAs were mentioned as a source of support by around 40% of women across all caste/tribal groups, with the exception of PVTGs (26%), but confidence intervals were wide. Poorer and less educated women were less likely to mention ASHAs as a source of support than better-off peers (no school vs. higher secondary+: adjusted RD: -16, 95%CI: -25 – -8; poorest vs. richest: adjusted RD: -23, 95%CI: -37 – -9).

## Discussion

Our study is, to our knowledge, the first to document the stillbirth rate and neonatal mortality rate among PVTGs in India. We demonstrate that NMRs are very high in this population, and contrast sharply with those in less disadvantaged groups. Deaths on the day of birth, deaths in the remainder of the early neonatal period, and late neonatal deaths contributed equally to these disparities. The higher neonatal mortality among PVTGs was only partly explained by their very high poverty levels and low maternal educational attainment; substantial mortality inequalities remained after adjustment for these factors. By contrast, SBRs were somewhat lower among PVTGs compared with other groups. The large disparities in neonatal mortality were mirrored by stark disparities in use of facility-based maternal and newborn health services. Again, these were partly explained by maternal education and wealth, but large inequalities remained after adjustment. Conversely, use of community-based services was more equitable.

We used data from one of the largest prospective demographic surveillance systems in a rural Indian state with a considerable tribal population. Nevertheless, our study had several limitations. First, the reported lower SBR among PVTGs is possibly due to misclassification between miscarriages (which were not included in the prospective surveillance system) and early stillbirths, as the much lower exposure to facility-based care among PVTGs might have made PVTG mothers less able to differentiate between these two outcomes, especially when the mother is not sure of her last menstrual period [29]. Yet, it is important to note that the stillbirth rate in our total study population, and across the sub-groups within that population, is much higher than the 10.6/1000 reported by Altijani et al. [11]. There may also have been misclassification between late stillbirths and early neonatal deaths, particularly for deaths on day 1. Unfortunately, no verbal autopsies were conducted. Yet, all deaths were cross-checked with information on crying and breathing at birth to minimize misclassification. There were six infants with an undetermined outcome. These were either recorded as stillbirth but with the infant breathing or crying after birth, or as neonatal death but with no breathing or crying. We excluded these deaths from our analyses. Our findings remained essentially the same in sensitivity analyses in which we either included these undetermined cases as stillbirths or as neonatal deaths. Secondly, our study area only comprised three out of Jharkhand’s 24 districts. Comparison with the National Family Health Survey (NFHS)-4 (2015-16) [30, 31], shows that the neonatal mortality levels are quite comparable to Jharkhand as a whole and that institutional delivery rates in our study population are comparable to those in rural areas of Jharkhand but the NFHS-4 also shows that inter-district variation is large. Uptake of the JSY scheme in our study population was much lower than in NFHS-4. A possible explanation for this low JSY uptake is a delay in receiving the actual cash, which means that some women might not have yet received cash from the JSY scheme at the time of the interview [32]. Finally, it is not possible to draw conclusions about women’s group attendance among PVTGs from our data, since there were only five births among PVTG-women in the intervention arm during the intervention period. Women’s group attendance was similar among ST-women compared with other women, but further research is needed to determine whether PVTGs are equally reached by this intervention.

A 2016 Lancet study on tribal and Indigenous health found that, worldwide, tribal populations often have poorer health and social outcomes [3]. Our findings support this conclusion. The Lancet study also revealed a paucity of reliable data on tribal health, especially in low- and middle-income countries. Such data are essential for documenting and monitoring health and social disparities between tribal peoples and other parts of the population, and can incite and underpin remedial policies and interventions. Our study documents SBRs and NMRs across tribal/caste groups in Jharkhand, using data from a population-based prospective demographic surveillance system. Our study shows there can be substantial inequalities in neonatal mortality among tribal populations too, as demonstrated by the higher neonatal mortality rates among PVTGs compared with other ST groups.

SBRs and NMRs among the extremely marginalised PVTG group have so far been undocumented. Their much higher levels of poverty and lower levels of maternal educational attainment partly explain their higher NMRs. Nevertheless, very large inequalities remain after these factors have been taken into account. Lack of access to facility-based maternal and newborn care is a known determinant of neonatal mortality [33, 34]. The huge gaps in use of such services between PVTGs and other groups will arguably partly explain the observed mortality inequalities. PVTGs have been pushed up the hills, to areas with low connectivity and long travel time to the district capital and other cities, whereas the OBC/none group tends to live in the lowlands, with better access to facilities, information and other resources that directly or indirectly influence neonatal health.

### Implications

Our study suggests several entry points for reducing the high neonatal mortality levels among PVTGs. The first entry point is addressing geographical barriers to facility-based maternal and newborn health services. Services that provide a ‘bridge’ between community and facilities already play a role here. For example, ASHAs accompany many PVTG women who want to deliver in health facilities. Moreover, maternity vehicles (provided by the facility, but called by the community) are frequently used among PVTG women who give birth at a facility. Use of both services is equitably distributed. Also, frontline health workers reach PVTG communities, and communicate in their own language. Nevertheless, this remains insufficient to close the gap in institutional births between PVTG and other groups. In addition, poorer and less educated women were less likely to report that they received support from an ASHA. It would be useful to examine why this is the case. Another important entry point for policy and intervention is action on the social determinants of tribal health. This includes improvement of educational facilities, provision of high-quality teachers, as well as a curriculum that is respectful of tribal cultures and languages and meaningful to PVTG families [35]. The government has set up welfare schemes for PVTGs in Jharkhand, including delivery of free rations at their doorstep. At the same time, it is also important to address the root causes of high levels of poverty among PVTGs, including forest depletion and displacement from their original lands. All such policies and interventions need to be based on the priorities and needs of PVTGs themselves and respect their rights and culture [4]. Finally, there is a high potential for community-based participatory learning and action interventions, which are based on local priorities and have facilitators grounded in local culture [36, 37]. The women’s group intervention has shown to be able to reduce neonatal mortality, including when facilitated by ASHAs. Our study shows a comparatively high uptake of this intervention across social groups, including STs.

At the same time, it is important to realize that PVTGs constitute a very small fraction of the population. Other ST, and SC groups also had higher mortality rates and lower uptake of many facility-based services compared with the OBC/none group. Even OBC are considered a deprived group. Furthermore, our paper reports on a systemic gradient in SBRs, neonatal mortality and facility-based services by maternal educational attainment and household wealth. These substantial inequalities are affecting a much larger part of the population. Thus, focusing solely on PVTG as the most marginalised group will not suffice to reduce neonatal mortality inequalities. Broader action is required to reduce health inequalities throughout the population, combined with targeted attention for the most marginalised groups, such as PVTG.

## Conclusions

Our study shows that socioeconomic inequalities in neonatal mortality in eastern India remain high and that PVTGs are most severely affected. Targeted interventions are required to reduce the very high neonatal mortality rates in PVTGs. Reducing geographical barriers to facility-based care and action on the broader social determinants of health to address the root causes of high levels of poverty and low educational attainment in these groups appear to be priorities. At the same time, policies and interventions should be based on the needs and priorities of PVTGs themselves and be respectful of their rights and culture. Interventions following principles of participatory learning and action with community-based groups have potential in this respect, but require further research in these highly marginalised groups.

It is important to combine these targeted interventions with action to address the broader health inequalities that affect large parts of society. While neonatal mortality inequalities between education and wealth groups are smaller in absolute terms than those between tribal/caste groups, their population health impact is larger because a much larger group is affected. Community-based interventions and services, such as women’s groups, and services that form a bridge between the community and facility, such as maternity vehicles and ASHA accompaniment to facilities, have potential to reduce these health inequalities.

## Supplementary Information

**Additional file 1.**

## Data Availability

The data will be made available through the UCL Discovery repository (https://discovery.ucl.ac.uk/) once the trial results are published.
