# Supplementary material for: Socioeconomic inequalities in stillbirth and neonatal mortality rates: evidence on Particularly Vulnerable Tribal Groups in eastern India
Source: Int J Equity Health. 2022 May 6;21:61. doi: 10.1186/s12939-022-01655-y (PMC9074184; doi:10.1186/s12939-022-01655-y)
Supplement: Supplementary file 1 — Additional file 1. [file 12939_2022_1655_MOESM1_ESM.docx]

**Supplementary Table 1: Number of live births, stillbirths and neonatal deaths in the study population**

|  | **Livebirths** | **Stillbirths** | **Neonatal deaths** | **Deaths on day 1** | **Deaths day 1-7** | **Deaths day 8-28** |
| --- | --- | --- | --- | --- | --- | --- |
| **Total population *** | 24660 | 567 | 966 | 417 | 776 | 190 |
|  |  |  |  |  |  |  |
| **Maternal tribe/caste^*^** |  |  |  |  |  |  |
| ST | 11981 | 251 | 542 | 213 | 424 | 118 |
| PVTG | 1015 | 18 | 60 | 24 | 44 | 16 |
| Other ST | 10966 | 233 | 482 | 189 | 380 | 102 |
| SC | 2953 | 85 | 124 | 66 | 104 | 20 |
| OBC/none | 9726 | 231 | 300 | 138 | 248 | 52 |
|  |  |  |  |  |  |  |
| **Maternal education** |  |  |  |  |  |  |
| Has not attended school | 7050 | 178 | 320 | 142 | 249 | 71 |
| Primary school | 7143 | 169 | 289 | 111 | 230 | 59 |
| Secondary school | 7297 | 145 | 248 | 112 | 209 | 39 |
| Higher secondary & above | 3110 | 62 | 102 | 47 | 81 | 21 |
|  |  |  |  |  |  |  |
| **Household wealth** |  |  |  |  |  |  |
| Poorest | 5602 | 148 | 262 | 99 | 200 | 62 |
| Next poor | 5352 | 147 | 254 | 116 | 202 | 52 |
| Middle poor | 5429 | 107 | 180 | 82 | 149 | 31 |
| Less poor | 4888 | 108 | 162 | 72 | 139 | 23 |
| Least poor | 3389 | 57 | 108 | 48 | 86 | 22 |

**^*^** Including singletons, first and second born of twins, and first, second and third born of triplets. As some women had twins/triplets, the 24,984 women in the control areas gave birth to 25,227 infants.

**^*^** ST = Scheduled Tribes, PVTG = Particularly Vulnerable Tribal Groups, Other ST = Other Scheduled Tribes, SC = Scheduled Caste, OBC/none = Other Backwards Class or other than the above

**Supplementary Table 2: Stillbirth rates and neonatal mortality rates by age group and parity**

|  | **Stillbirths**  Rate per 1000 births | **Neonatal mortality** rate per 1000 live births | **Mortality on day 1** rate per 1000 live births | **Early neonatal mortality (day 1-7)** rate per 1000 live births | **Late neonatal mortality (day 8-28)** rate per 1000 live births |
| --- | --- | --- | --- | --- | --- |
|  |  |  |  |  |  |
| **Total population** | 22 | 39 | 17 | 31 | 8 |
|  |  |  |  |  |  |
| **Age** |  |  |  |  |  |
| < 20 years | 33 | 62 | 27 | 52 | 10 |
| 20-24 years | 19 | 36 | 16 | 29 | 7 |
| 25-29 years | 20 | 31 | 13 | 24 | 7 |
| 30-34 years | 24 | 50 | 18 | 38 | 11 |
| 35+ years | 43 | 41 | 21 | 30 | 11 |
|  |  |  |  |  |  |
| **Parity** |  |  |  |  |  |
| Primiparity | 25 | 51 | 21 | 42 | 9 |
| Multiparity | 21 | 34 | 15 | 26 | 7 |
|  |  |  |  |  |  |
